# The interplay between lipid metabolism, cancer progression and anti-cancer immunity

**DOI:** 10.1038/s12276-026-01783-3

**Published:** 2026-08-18

**Authors:** Choong-Hyun Koh, Yoosun Lee, Il-Kyu Kim, Yeonseok Chung

**Affiliations:** 1 Laboratory of Immune Regulation, Research Institute of Pharmaceutical Sciences, Seoul, South Korea; 2 The Center for Viral Immunology, Korea Virus Research Institute, Institute for Basic Science (IBS), Daejeon, 34126 Republic of Korea; 3 College of Pharmacy, Gachon University, Incheon, 21936 Republic of Korea

**Keywords:** Cancer metabolism, Tumour immunology, Cancer microenvironment

## Abstract

The tumour microenvironment (TME) consists of a complex ecosystem of tumour cells, stromal elements, extracellular matrix, fibroblasts and diverse immune-cell populations. Although traditional cancer therapies have primarily focused on eliminating tumour cells, subsequent advances highlighted the importance of modulating immune cells within the TME to enhance anti-tumour immunity. More recently, a major conceptual shift has emerged: targeting nutrient metabolism as a therapeutic strategy. Because tumours create a hypoglycaemic, metabolically restrictive niche, both cancer cells and infiltrating immune cells increasingly rely on lipid uptake, storage and catabolism to sustain their survival and function. This metabolic dependency reveals new vulnerabilities that can be exploited for cancer therapy. In this review, we integrate fundamental concepts of lipid metabolism with recent mechanistic discoveries that define how lipids shape cellular behaviours within the TME, emphasizing the dual roles of lipids as both metabolic substrates and signalling mediators. We also highlight emerging evidence demonstrating that dietary lipid composition — beyond total fat intake — profoundly influences tumour progression and anti-tumour immunity. Together, these insights position lipid metabolism as a critical regulator of tumour biology and an attractive target for next-generation cancer therapies.

## Lipid Metabolism Shapes the Tumour Microenvironment and Immunity

Lipid metabolism plays a crucial role in cancer progression. This review explores how cancer cells adapt their lipid metabolism to thrive in nutrient-deprived environments, enhancing fatty-acid oxidation and lipid synthesis to support rapid proliferation. The authors discuss the impact of dietary fatty acids on tumour progression, highlighting how specific fats can modulate anti-tumour immunity. They utilize a comprehensive analysis of lipid metabolic pathways, focusing on enzymes such as fatty acid synthase and carnitine palmitoyltransferase 1, which are upregulated in various cancers. The review reveals that targeting these pathways can inhibit tumour growth, as demonstrated by the effectiveness of fatty-acid synthase inhibitors in preclinical models. The study underscores the potential of dietary interventions and metabolic therapies in cancer treatment, suggesting that future research should focus on the interplay between diet, lipid metabolism and cancer to develop effective therapeutic strategies.

This summary was initially drafted using artificial intelligence, then revised and fact-checked by the author.

## Introduction

Lipids are essential nutrients, functioning as energy sources, structural components of cellular membranes and substrates for signalling molecules^1,2^. Lipid metabolic reprogramming has emerged as a hallmark of cancer, supporting elevated bioenergetic demands required for sustained proliferation. To thrive in both lipid-enriched and lipid-deprived tumour microenvironments (TMEs), cancer cells enhance lipid uptake and activate de novo lipid synthesis^3–5^. Cholesterol, glycolipids and phospholipids form the structural core of cellular membranes, with cholesterol playing an additional role in organizing signalling platforms within lipid rafts. Elevated cholesterol levels — whether imported or synthesized via the mevalonate pathway — frequently accumulate in tumours and promote survival and growth^6^. In addition, lipid mediators generated by phospholipase-dependent cleavage of membrane lipids or by endogenous modification of dietary fatty acids (FAs) act as potent signalling molecules that further support tumour growth^7^. Lipid metabolic rewiring is shaped not only by cancer-cell-intrinsic oncogenic programmes but also by dynamic crosstalk with cells in the TME, ultimately influencing the cellular composition in the TME^8^. As such, lipid metabolism is increasingly recognized as a central driver of cancer progression and a promising therapeutic target.

Tumour initiation, progression and therapeutic responses are regulated by both cancer-cell-intrinsic and -extrinsic factors, including host immune system and microbiota^9–11^. Diet has recently emerged as an additional layer of regulation, and a conceptual framework has been proposed to dissect how diet shapes tumour growth^12,13^. Alterations in dietary composition change the availability of specific nutrients that cancer cells exploit, thereby influencing their survival and growth. Consistent with this, obesity is associated with poor clinical outcomes in patients with breast and colon cancer^14^. High-fat diets (HFDs) increase the incidence and growth of multiple cancer types in preclinical models through cellular and molecular mechanisms that remodel cancer-cell phenotypes and diverse populations within the TME^15–20^, suggesting the pro-tumorigenic role of dietary lipids. More recently, the composition of dietary FAs — saturated, monounsaturated FA (MUFA) and polyunsaturated FA (PUFA) — has been shown to differentially modulate anti-tumour immunity^21^.

In this review, we summarize the landscape of lipid metabolism within the TME, discuss the impact of specific dietary FAs on tumour progression and highlight emerging therapeutic strategies that target key molecular regulators of lipid metabolic rewiring in cancer cells, as well as approaches to modulating lipid metabolism in the TME through dietary interventions.

## Lipid metabolism in cancer cells

**Fig. 1:**
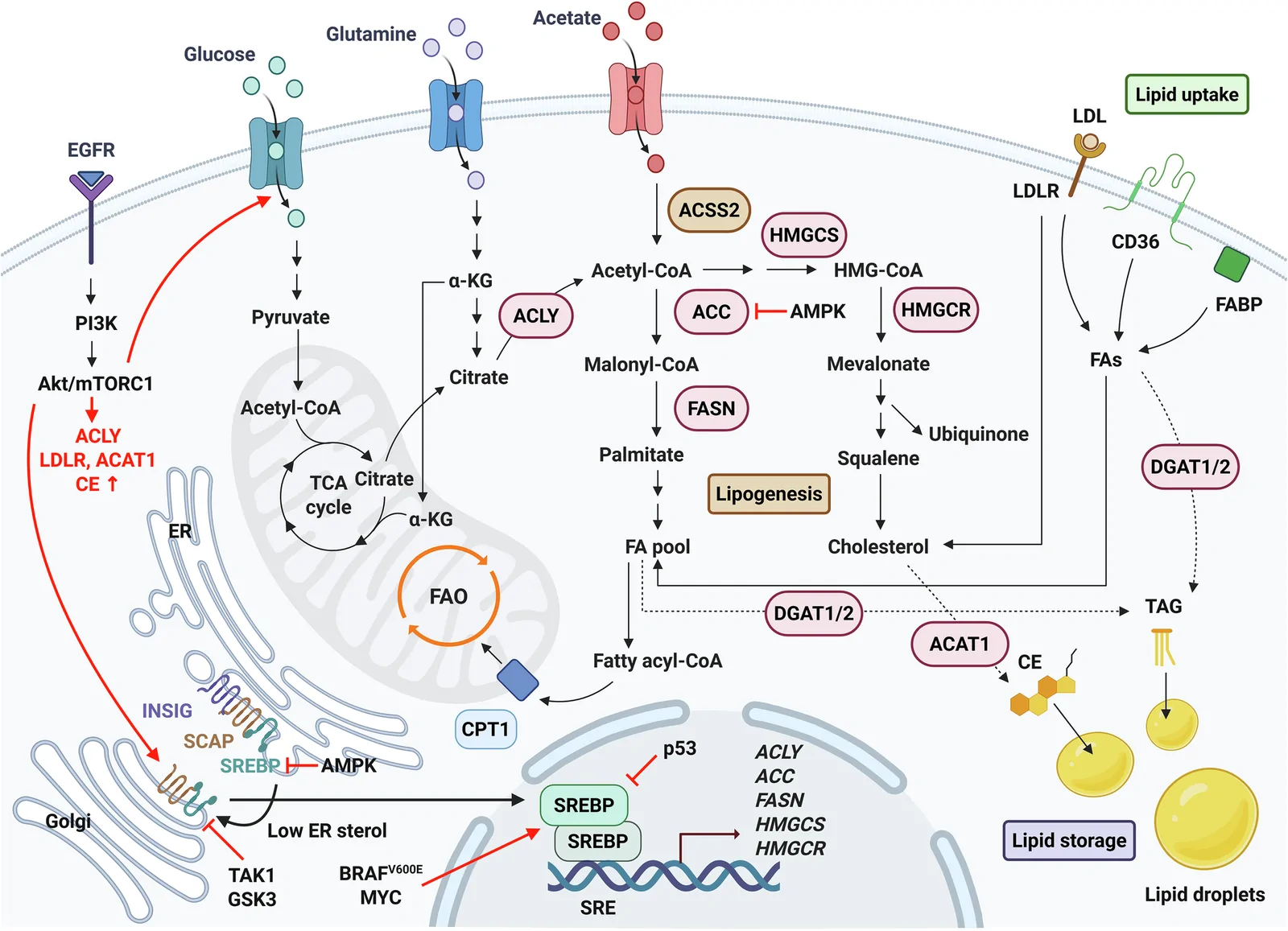
Lipid metabolic rewiring in cancer: pathways, regulators and therapeutic vulnerabilities. Cancer cells reprogram lipid metabolism by enhancing fatty-acid oxidation (FAO), de novo lipid synthesis, lipid uptake and storage, thereby sustaining growth and metastatic potential. FAO supplies acetyl-CoA, with CPT1 and PPARs serving as key regulators. Lipogenesis is fuelled by acetyl-CoA generated from glucose-, glutamine- or acetate-derived pathways via ACLY and ACSS2, followed by ACC- and FASN-mediated synthesis of fatty acids. Cholesterol biosynthesis through the mevalonate pathway, with HMGCR being a rate-limiting enzyme, further supports membrane production, redox balance and tumour survival. Under low ER sterol conditions, the SCAP–SREBP complex dissociates from INSIG and is transported to the Golgi, where SREBPs undergo proteolytic cleavage. The resulting active forms translocate to the nucleus to induce genes required for fatty-acid and cholesterol biosynthesis. Lipid uptake is mediated by receptors such as CD36, FABP and LDLR, whereas DGAT1/2 and ACAT1 enable lipid droplet (LD) formation to buffer lipotoxicity. Multiple oncogenic pathways — including EGFR, PI3K–Akt, mTORC1, BRAF and MYC — converge on SREBP activation, upregulating lipogenic enzymes and promoting tumour growth. In contrast, AMPK, TAK1, GSK3 and tumour suppressor p53 restrain lipogenesis by repressing SREBP or ACC activity. α-KG, α-ketoglutarate; ACAT1, acyl-CoA:cholesterol acyltransferase 1; ACC, acetyl-CoA carboxylase; ACLY, ATP-citrate lyase; ACSS2, acetyl-CoA synthetase 2; CE, cholesterol ester; CPT1, carnitine palmitoyltransferase 1; DGAT1/2, diglyceride acyltransferase 1/2; FABP, fatty-acid-binding protein; FASN, fatty-acid synthase; GSK3, glycogen synthase kinase 3; HMGCR, hydroxy-3-methylglutaryl-CoA reductase; HMGCS, 3-hydroxy-3-methylglutaryl-CoA synthase; INSIG, insulin-induced gene; LDLR, low-density lipoprotein receptor; SCAP, SREBP cleavage-activating protein; SREBP, sterol regulatory element-binding protein; TAG, triacylglycerol; TAK1, transforming growth factor-β activated kinase 1.

As tumours progress, they are frequently exposed to nutrient-deprived conditions characterized by limited glucose and glutamine availability but increased lipid accumulation^22^. To adapt, cancer cells reprogram lipid metabolism by upregulating genes involved in FA oxidation (FAO, also called β-oxidation), de novo lipogenesis, lipid uptake and storage (Fig. 1), thereby sustaining tumour-cell growth and enhancing their metastatic potential. Consequently, therapeutic strategies targeting lipid metabolic enzymes and pathways in cancer cells are emerging as a promising approach to cancer treatment.

### Lipid metabolic reprogramming and functional consequences in cancer

#### Fatty-acid oxidation and lipogenesis

Utilization of fat as an energy source begins with two sequential steps: lipolysis, which hydrolyses triacylglycerol (TAG) into FAs and glycerol, followed by FAO. FAO generates acetyl-CoA to fuel the TCA cycle, as well as NADH and FADH_2_ produced during both FAO and the TCA cycle, thereby driving mitochondrial electron transport chain-mediated ATP production. Numerous enzymes and transporters participate in this process, and their elevated expression has been reported across various cancer types and associated with tumour progression^23^.

In the initial step, long-chain FAs (LCFAs) are converted to fatty acyl-CoA by fatty acyl-CoA synthetase. Carnitine palmitoyltransferase 1 (CPT1) then catalyses the formation of fatty acylcarnitine, which is transported into the mitochondrial matrix via carnitine acylcarnitine translocase (CACT). CPT1C is highly expressed in human lung cancer and confers resistance to glucose-depleted conditions by promoting FAO^24^. Notably, etomoxir, a CPT1 inhibitor, blocks FAO and sensitizes leukaemia cells to pro-apoptotic agents^25^. Within the mitochondrial matrix, CPT2 converts fatty acylcarnitine back to fatty acyl-CoA, which subsequently undergoes FAO to generate acetyl-CoA, thereby supporting the TCA cycle and ATP production. Peroxisome proliferator-activated receptors (PPARs) serve as key regulators of FAO, sensing FA-derived signals and controlling the transcription of enzymes involved in this metabolic pathway^26,27^.

Cancer cells reactivate de novo lipogenic pathways to supply the biomass and bioenergetic substrates required for rapid proliferation. Cytoplasmic acetyl-CoA is a central metabolite in lipid synthesis and can be generated from glucose, glutamine or acetate^28^. Glucose-derived pyruvate enters the mitochondrial TCA cycle and produces citrate, which is exported to the cytosol and converted into acetyl-CoA by ATP-citrate lyase (ACLY). Under hypoxic conditions or in cells with impaired mitochondria function, tumour cells generate citrate from glutamine-derived α-ketoglutarate via reductive carboxylation^29,30^. Acetate also serves as a substrate for acetyl-CoA production through the action of cytosolic acetyl-CoA synthetase 2 (ACSS2). Both ACLY and ACSS2 are upregulated in multiple cancers and contribute to tumour growth by supporting lipogenesis, as well as promoting histone acetylation-mediated transcription programmes associated with autophagy and cell survival^31–34^. Acetyl-CoA carboxylase (ACC) then catalyses the conversion of acetyl-CoA to malonyl CoA, which is subsequently utilized by fatty-acid synthase (FASN) to produce saturated FA palmitate. The anti-tumour effects of ACC inhibitors have been demonstrated in xenograft models^35,36^. Inhibition of FASN induces tumour-cell apoptosis by blocking FA synthesis and causing accumulation of malonyl-CoA, which in turn suppresses FAO. FASN inhibitors have shown promising anti-tumour activity in preclinical models, and clinical trials are currently underway^23,37,38^.

In lipid-poor environments such as the brain, metastatic cancer cells adapt by upregulating enzymes involved in de novo fatty acid synthesis, creating a site-specific vulnerability, as genetic or pharmacological inhibition of FASN effectively suppresses breast tumour metastasis in the brain^39^. Beyond tissue context, systemic interventions such as caloric restriction (CR) suppress tumour growth by limiting lipid desaturation through stearoyl-CoA desaturase (SCD) inhibition, thereby disrupting the balance between MUFA and saturated fatty acids (SFA) required for membrane biosynthesis and proliferation, thereby limiting tumour growth^40^. Enforcing cancer-cell SCD expression or increasing circulating lipids through a high-fat CR diet partially rescues the growth inhibition caused by CR. These findings suggest that dietary fat composition plays a significant role in human tumour growth and identifying diets that induce lipid depletion or SFA accumulation could improve the efficacy of cancer therapies, which will be discussed later. Furthermore, some cancer cells exhibit metabolic plasticity by engaging alternative desaturation pathways, such as fatty acid desaturase 2 (FADS2)-mediated sapienate production^41^, thereby bypassing canonical SCD dependency and presenting a potential avenue for therapeutic intervention.

Cholesterol, together with phospholipids and glycolipids, is a major component of biological membranes and is synthesized from acetyl-CoA via the mevalonate pathway. In this pathway, three molecules of acetyl-CoA are condensed by 3-hydroxy-3-methylglutaryl (HMG)-CoA synthase (HMGCS) to form HMG-CoA, which is subsequently converted to mevalonate by HMG-CoA reductase (HMGCR), the rate-limiting enzyme of the pathway. Inhibition of HMGCR by statins sensitizes blood-cancer cells to the pro-apoptotic drug venetoclax and has been evaluated in clinical trials^42,43^. Intermediate metabolites of cholesterol biosynthesis, such as ubiquinone and squalene, contribute to redox control and support cancer-cell survival under oxidative stress in colorectal and pancreatic cancers^44,45^.

Sterol regulatory element-binding proteins (SREBPs) are a family of transcription factors that play a central role in lipogenesis by upregulating the expression of enzymes and transporters required for FA and cholesterol synthesis^46–48^ (Fig. 1). These lipogenic proteins comprise three isoforms: SREBP1a and SREBP1c (encoded by *SREBF1*), which primarily regulate genes involved in FA synthesis, and SREBP2 (encoded by *SREBF2*), which preferentially controls genes associated with cholesterol biosynthesis^46,49^. Inactive SREBPs reside in the endoplasmic reticulum (ER) as membrane-bound proteins, where they form a complex with SREBP cleavage-activating protein (SCAP) and insulin-induced gene (INSIG). When ER sterol levels fall, SCAP dissociates from INSIG and interacts with coatomer II (COPII) vesicles, leading to the translocation of the SCAP–SREBP complex from the ER to the Golgi. There, mature SREBPs are generated through sequential proteolytic cleavages. The active SREBPs then translocate into the nucleus and transactivate their target genes involved in lipid biosynthesis by binding to the SRE motif in the promoter regions^1,50,51^. Accumulating evidence indicates that active SREBP1 is elevated in multiple cancer types and contributes to tumour progression and metastasis^52–55^.

#### Lipid uptake and storage in lipid droplets

Although cancer cells can be fuelled by themselves through de novo lipogenesis machinery, they also acquire FA and cholesterol from extracellular milieu via receptor- and binding protein-mediated lipid uptake. CD36, also known as FA translocase, is a scavenger receptor that imports extracellular lipids and has been associated with poor prognosis across multiple cancer types^56^. Indeed, gene deletion or therapeutic blockade of CD36 has been shown to reduce tumorigenesis and metastasis in several preclinical cancer models by limiting FA uptake in cancer cells^57–59^. Moreover, the expression of FA-binding proteins (FABPs) is upregulated in metastatic ovarian cancer cells, facilitating lipolysis in adjacent adipocytes and the subsequent release of free FAs. These FAs are then taken up by cancer cells through FABP4, thereby promoting metastatic tumour growth^60^. Several studies have also demonstrated that low-density lipoprotein receptors (LDLRs) contribute to cholesterol uptake and tumour growth, and their elevated expression is associated with poor prognosis in lung, breast and pancreatic cancers^61–63^.

Multiple cancer types have been shown to store excess lipids in lipid droplets (LDs) in the form of TAG and cholesterol ester (CE), both as a reserve fuel source and as a means of preventing lipotoxicity^64^. In this process, the enzymes diglyceride acyltransferase 1/2 (DGAT1/2) and acyl-CoA:cholesterol acyltransferase 1 (ACAT1, also known as sterol O-acyltransferase 1 (SOAT1)) catalyse the conversion of diacylglycerol to TAG and cholesterol to CE, respectively. DGAT1 expression is upregulated in glioblastoma and clear-cell renal-cell carcinoma (ccRCC), and DGAT1 inhibition increases ROS production and apoptosis, thereby impairing tumour growth^65,66^. Similarly, deletion of ACAT1 suppresses cancer growth by reducing LD formation and lipogenesis^67,68^. Collectively, cancer cells develop diverse molecular mechanisms that enable them to accumulate excess lipids while evading lipotoxicity, ultimately supporting their rapid growth and survival.

#### Lipid metabolism shapes responses to immunotherapy

Tumour cell-intrinsic lipid metabolic programmes have emerged as critical, yet context-dependent, determinants of the response to cancer immunotherapy. Accumulating evidence indicates that de novo lipogenesis and cholesterol-related anabolic pathways in multiple cancers not only sustain tumour growth under nutrient-limited conditions but also drive immune evasion. For instance, elevated FASN expression correlates inversely with immunotherapy response signatures, including T cell infiltration, cytolytic activity and HLA-I expression, whereas its depletion enhances T cell-mediated killing and suppresses PD-L1 induction, highlighting FASN as a metabolic checkpoint that can be targeted to improve the efficacy of immunotherapy^69^. Similarly, nucleo-cytosolic acetyl-CoA promotes immune escape through epigenetic upregulation of PD-L1 via P300-dependent histone H3K27 acetylation^70^, with targeting of acetyl-CoA-producing enzyme ACLY improving the response to immune checkpoint inhibitors.

Cancer lipid metabolism also affects whether cancer cells are physically vulnerable to immune killing. CD8 T cell-derived IFNγ, together with arachidonic acid, enhances acyl-CoA synthetase long-chain family member 4 (ACSL4)-dependent incorporation of polyunsaturated FAs into membrane phospholipids, thereby sensitizing tumour cells to ferroptosis and augmenting anti-tumour immunity^71^. Consistently, loss of ACSL4 impairs T cell-mediated tumour control, whereas ferroptosis induction enhances checkpoint therapy efficacy. Furthermore, ACSL4 correlates positively with the response to adoptive T cell therapy in melanoma patients. T cell-intrinsic lipid metabolism in cancer immunity is bidirectional, either sustaining anti-tumour persistence and function or, if dysregulated, contributing to metabolic insufficiency and exhaustion, which will be discussed later. Therefore, these findings provide a strong rationale for combinatorial strategies that simultaneously disrupt tumour lipid dependencies while enhancing T cell metabolic fitness to maximize immunotherapy efficacy.

### Crosstalk between lipid metabolic pathways and oncogenes/tumour suppressor genes

Oncogenic signalling pathways have been reported to support cancer-cell growth and survival by upregulating SREBP activity and lipogenesis^2^ (Fig. 1). Epidermal growth factor receptor (EGFR) signalling increases glucose uptake and SCAP N-glycosylation, which in turn facilitates trafficking and activation of the SCAP–SREBP complex to promote lipogenesis and cell growth in glioblastoma, thereby demonstrating the molecular link between glucose availability and lipid biosynthesis^23,72^. Guo et al. also showed that EGFR-activated glioblastomas induce the nuclear translocation of SREBP1 through PI3K–Akt signalling and exhibit therapeutic vulnerability to FA and cholesterol inhibitors^53^.

Akt activation in hepatocellular carcinoma (HCC) has been shown to phosphorylate the gluconeogenic enzyme phosphoenolpyruvate carboxykinase 1 (PCK1), which in turn phosphorylates INSIG and releases the SCAP–SREBP complex to the Golgi, thereby activating SREBPs. Consequently, this Akt–PCK1–INSIG axis accelerates xenograft liver tumour growth, and phosphorylated PCK1 and INSIG1/2 are associated with poor prognosis in patients with HCC^73^. In addition, oncogenic Akt enhances ACLY activation, leading to increased production of acetyl-CoA, which supports histone acetylation and fuels the mevalonate pathway, thereby contributing to pancreatic tumorigenesis^74,75^. Furthermore, PTEN loss and subsequent activation of the PI3K–Akt pathway drive aberrant accumulation of cholesteryl ester within LDs in prostate cancer, primarily by augmenting low-density lipoprotein (LDL) uptake and the activity of cholesterol-esterifying enzyme ACAT1. Suppression of CE levels through pharmacological or genetic inhibition of ACAT1 markedly diminishes the survival, migratory and invasive capacities of prostate-cancer cells, and impairs the growth of xenograft tumours^67^. The role of mTORC1 in promoting SREBP activation is also well established and is mediated, at least in part, through the suppression of autophagy and the consequent reduction of ER sterol levels^76–78^.

It has also been demonstrated that mutant BRAF drives SREBP1-mediated lipogenesis in melanoma, and that tumour cells resistant to BRAF-targeted therapy, vemurafenib, maintain SREBP1 activity, thereby protecting themselves from lipid peroxidation as part of therapy resistance mechanisms. Accordingly, the study showed that co-administration of SREBP1 inhibitors represents a viable strategy for enhancing therapy response to vemurafenib in melanoma^79^.

In addition, proto-oncogene Myc has been shown to transcriptionally cooperate with MondoA, a nutrient-sensing member of the Myc superfamily, to regulate lipid metabolic programmes that support cell survival and proliferation in neuroblastoma^80^. This study further demonstrated that genes co-regulated by Myc and MondoA are associated with poor clinical outcomes across multiple cancer types. Moreover, Myc has been shown to induce and collaborate with SREBP1 to enhance lipogenesis during tumorigenesis, thereby conferring therapeutic vulnerability of Myc-driven tumours to the inhibition of FA synthesis^81^.

Tumour suppressor p53 induces the expression of the cholesterol transporter gene ATP-binding cassette transporter (ABCA1) and sustains ER sterol levels, thereby preventing SREBP2 activation and suppressing liver tumorigenesis. Conversely, derepression of the mevalonate pathway or suppression of ABCA1 is required for HCC development, as demonstrated by genetic or pharmacological inhibition of mevalonate pathway-related genes and loss-of-function of ABCA1 (ref. ^82^).

Moreover, activation of AMP-activated protein kinase (AMPK) phosphorylates and inhibits lipogenic functions of ACC and SREBPs, thereby suppressing the development of HCC^83,84^. However, AMPK-mediated inhibition of ACC also exhibits pleiotropic effects, as it can accelerate solid tumour growth by enhancing NADPH-mediated redox control and promoting cancer-cell survival^85^. Similar to AMPK, transforming growth factor-β activated kinase 1 (TAK1) phosphorylates and inhibits SREBPs, thereby preventing HCC progression^86^. Glycogen synthase kinase 3 (GSK3) is also known to induce phosphorylation-dependent, ubiquitin-mediated degradation of SREBPs in various cancer-cell lines. This effect is counteracted by mTORC2 and protein arginine methyltransferase 5 (PRMT5), which inhibit SREBP phosphorylation and are associated with poor prognosis of HCC^87–89^.

The lipogenic functions of liver X receptors (LXRs), LXRα and LXRβ, are well-documented in cancer^90^. In the presence of LXR ligands such as oxysterols, LXRs form heterodimers with the retinoid X receptor (RXR), bind to LXR response elements (LXREs) in the promoters of multiple genes involved in lipogenesis and cholesterol transport, and subsequently induce their expression. Consistent with these roles, an LXR antagonist has demonstrated broad anti-tumour activity across diverse cancer types^91^. However, LXR agonists have also been shown to suppress glioblastoma, in part because of cancer-cell-type-specific metabolic dependency on cholesterol metabolism and context-dependent expression of genes associated with cholesterol uptake and transport^92,93^.

### Crosstalk between cancer cells and the tumour microenvironment via altered lipid metabolism

Lipid mediators generated by membrane phospholipases (PLA, PLC and PLD) play essential roles in both intracellular and intercellular signalling^7^. Among these enzymes, PLA2 cleaves membrane phospholipids to yield arachidonic acid (AA) and the signalling lipid lysophosphatidic acid (LPA). AA is further metabolized into prostaglandins (PGs) and leukotrienes through the enzymatic actions of cyclooxygenases (COX) and lipoxygenases (LOX), respectively. These downstream metabolites exert potent pro-inflammatory and immunosuppressive effects^94^. For instance, tumour-derived PGE_2_ has been shown to orchestrate the formation of the immunosuppressive TME by inducing IL-6, CXCL1, G-CSF, GM-CSF and adenosine, thereby promoting tumour progression^95,96^.

Obesity and elevated cholesterol levels are associated with increased recurrence and poor overall survival in patients with breast cancer. Baek et al. demonstrated that the oxysterol metabolite 27-hydroxycholesterol mediates the pro-metastatic effects of the HFD by promoting the accumulation of immature monocytes, neutrophils and γδ T cells, while reducing cytotoxic CD8^+^ T cells at both primary and metastatic sites^97^. Accordingly, genetic or pharmacological inhibition of CYP27A1, the enzyme responsible for 27-hydroxycholesterol biosynthesis, significantly reduces tumour metastasis in mouse models of breast cancer. Furthermore, cholesterol enrichment within the TME has been shown to upregulate the expression of immune checkpoint including PD-1, TIM-3, 2B4 and LAG-3 on tumour-infiltrating CD8^+^ T cells through ER-stress-induced activation of XBP1, thereby diminishing anti-tumour activity^98^. Collectively, these findings demonstrate that cholesterol and its metabolite exert pro-tumorigenic effects in a cancer-cell-extrinsic manner by reshaping the composition and functional state of tumour-infiltrating immune cells.

**Fig. 2:**
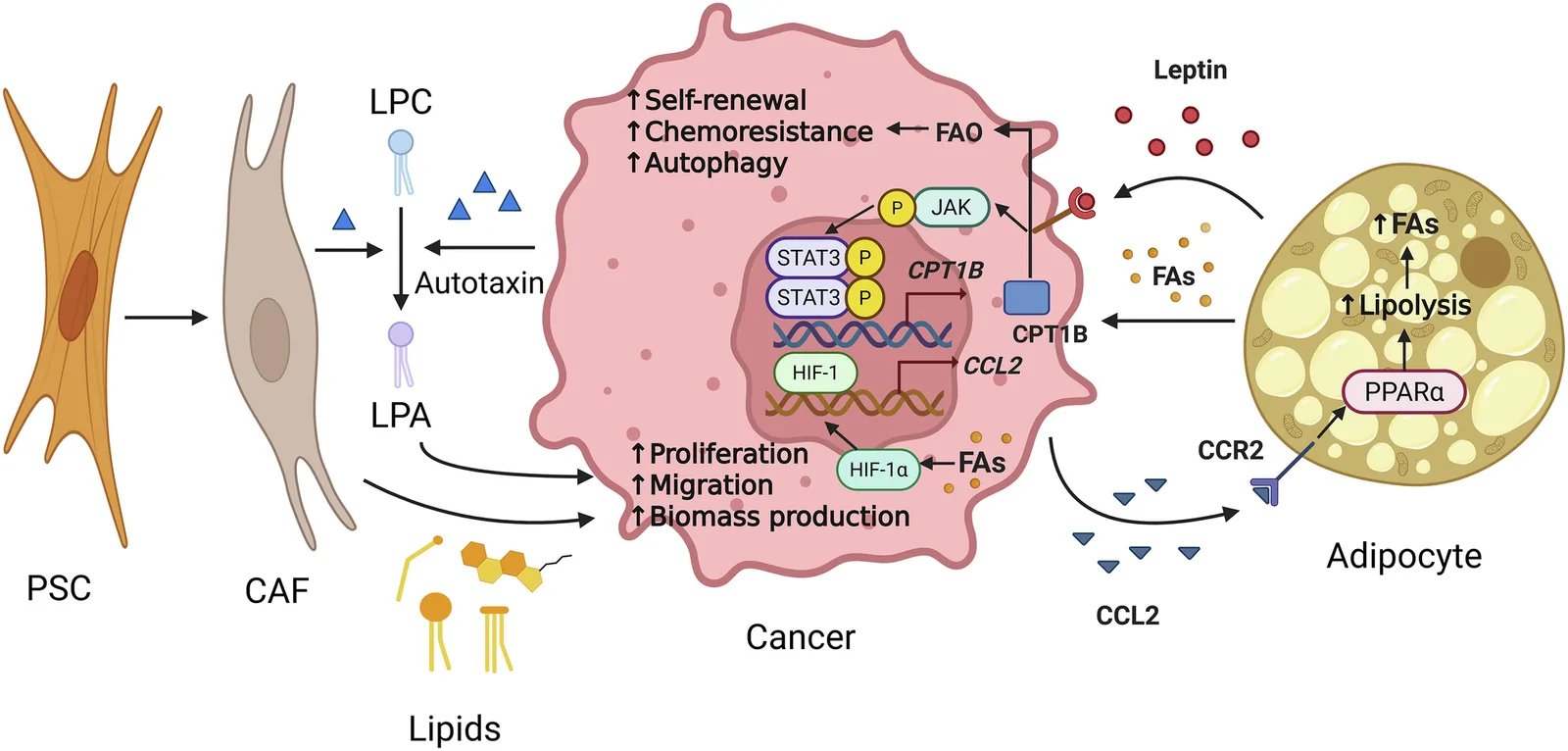
Lipid metabolic crosstalk between cancer and stromal cells/adipocytes. During pancreatic tumorigenesis, pancreatic stellate cells (PSCs) transdifferentiate into cancer-associated fibroblasts (CAFs) and undergo marked lipidome remodelling, leading to the secretion of lysolipids that fuel pancreatic ductal adenocarcinoma (PDAC) growth through autotaxin-dependent production of bioactive lipids. In breast cancer, mammary adipocytes secrete leptin, which enhances fatty-acid oxidation (FAO) in cancer stem cells via JAK/STAT3-mediated induction of CPT1B, whereas adipocyte-derived fatty acids (FAs) further support stemness and chemoresistance. Conversely, cancer cells can induce lipolysis in adipocytes, thereby promoting metastatic progression in ovarian cancer and fostering a chemoresistant niche for CD36^+^ leukaemic stem cells. This reverse metabolic flux is mediated in part by an HIF-1α–CCL2 axis that activates PPARα signalling in adipocytes. LPA, lysophosphatidic acid; LPC, lysophosphatidicholine.

Pancreatic ductal adenocarcinoma (PDAC) is characterized by a pronounced stromal reaction, fulfilled in part by cancer-associated fibroblasts (CAFs). During pancreatic tumorigenesis, pancreatic stellate cells can transdifferentiate into CAFs and undergo dramatic remodelling of their intracellular lipidome. These CAFs subsequently secrete specific lysolipids in the TME, which fuels PDAC by promoting biomass synthesis and generating signalling molecules through the enzyme autotaxin (Fig. 2), clearly demonstrating metabolic coupling between cancer cells and stroma and offering a promising therapeutic opportunity^99^.

Mammary adipocytes secrete leptin, which promotes FAO in breast-cancer stem cells through JAK/STAT3-mediated upregulation of CPT1B (Fig. 2). In addition, adipocyte-derived FAs can fuel breast cancer, thereby enhancing cancer-cell stemness and contributing to chemoresistance^100^. Similarly, colon cancer cells take up free FAs released from adipocytes and acquire a survival advantage under nutrient-deprived conditions, in part through AMPK activation-mediated induction of autophagy^101^. In addition, co-culture with adipocytes increases the expression of genes associated with cancer stemness while decreasing the expression of genes linked to epithelial cell differentiation in a 3D tumour organoid model. Consistently, colorectal-cancer cells transplanted with adipocytes exhibit enhanced tumour growth in a xenograft model, demonstrating the pro-tumorigenic effect of adipocytes. Conversely, cancer-cell-induced lipolysis in adipocytes has been shown to facilitate metastatic tumour growth in ovarian cancer and to create a supportive niche for chemoresistance in CD36^+^ leukaemic stem cells^60,102^. This process is mediated, at least in part, by a cancer-cell hypoxia-inducible factor-1α (HIF-1α)–CCL2 axis that activates PPARα signalling in adipocytes^20^, further highlighting the bidirectional metabolic crosstalk between adipocytes and cancer cells (Fig. 2).

## Lipids and immune cells

Immune cells are critical regulators of tumour growth within the TME. However, the immunosuppressive nature of the TME — driven by hypoxia, acidosis and inhibitory molecules expressed by suppressive populations such as regulatory T cells (Tregs), tumour-associated macrophages (TAMs) and tumour cells — induces functional impairment in anti-tumour immunity. During tumour progression, the TME becomes enriched with a heterogeneous pool of lipids (including FAs, cholesterol and oxidized lipids) that further reinforces immunosuppression^18,98^. Under such conditions, immune cells accumulate lipids in LDs, catabolizing them through FAO when needed. This process is accompanied by metabolic reprogramming regulated by lipid-metabolic pathways, including the PPAR family and SREBP, which can ultimately lead to either immune dysfunction or, in some contexts, enhanced immune activity.

### CD8^+^ T cells

Effector CD8^+^ T cells primarily rely on glycolysis to sustain rapid proliferation and cytotoxic activity, whereas memory CD8^+^ T cells mainly preferentially utilize energy-efficient processes such as OXPHOS and FAO to support survival^103^. As tumours progress and the microenvironment becomes increasingly hypoglycaemic and hypoxic, CD8^+^ T cells undergo metabolic reprogramming characterized by enhanced lipid uptake and activation of FAO through PPARα signalling, enabling the use of fatty acids as an alternative energy source^22^.

**Fig. 3:**
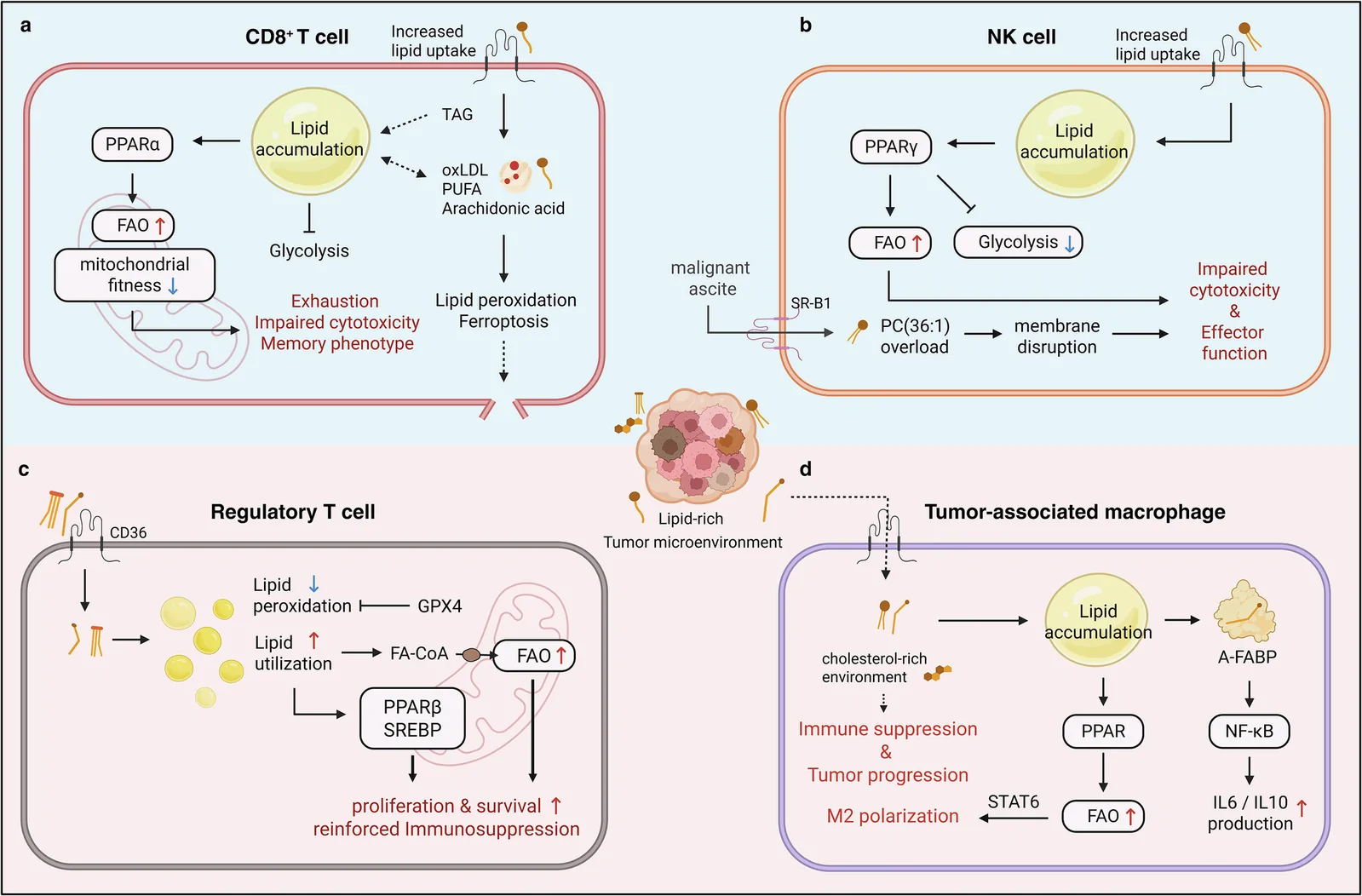
Metabolic reprogramming of immune cells within the tumour microenvironment. During tumour progression, the tumour microenvironment (TME) becomes enriched with heterogeneous lipid species, which collectively reinforce immunosuppression. In this lipid-rich condition, CD8^+^ T cells (**a**) and natural-killer (NK) cells (**b**) exhibit reduced effector function owing to increased lipid uptake and intracellular lipid accumulation, which activate peroxisome proliferator-activated receptors (PPARs) and promote fatty acid oxidation (FAO). This metabolic shift is associated with T cell exhaustion, impaired cytotoxicity and a dysfunctional memory-like phenotype. Suggested mechanisms include lipid peroxidation and ferroptosis driven by accumulated oxidized low-density lipoprotein (oxLDL), polyunsaturated fatty acids (PUFAs) and arachidonic acid; impaired mitochondrial fitness; and membrane disruption caused by excessive lipid accumulation. In contrast, Tregs (**c**) utilize FAO to support their proliferation and survival, whereas activation of PPARβ and SREBP pathways enhances their immunosuppressive function. Tregs also upregulate GPX4 expression to prevent lipid peroxidation-induced ferroptosis. Tumour-associated macrophages (**d**) undergo FAO-driven M2 polarization, and A-FABP facilitates NFκB-mediated production of IL-6 and IL-10, thereby promoting tumour progression. This figure highlights predominant immunosuppressive lipid metabolic pathways, whereas adaptive or context-dependent effects are discussed in the main text.

Importantly, lipid-driven metabolic reprogramming in CD8^+^ T cells can be either adaptive or pathological, depending on the cellular context and lipid composition of the TME. In adaptive settings, FAO supports the activation, survival and memory differentiation of CD8^+^ T cells. CD8^+^ tissue-resident memory T cells (Trm), which play a critical role in anti-tumour immunity and suppression of metastasis in adenocarcinoma, are highly dependent on FAO for their activation and survival^104^ (Fig. 3). In line with the emerging concept of nutrients as a “signal 4” in T cell biology, specific lipid species can directly enhance CD8^+^ T cell fitness. Signal 4 refers to nutrients — such as glucose, amino acids and lipids — that function as an essential fourth activation cue for T cells by driving nutrient transport, sensing and metabolic signalling to license full T cell activation, differentiation and effector function^105^. During CD8^+^ T cell activation, PUFAs are consumed to a greater extent than MUFAs or SFAs, and among them, linoleic acid (18:2, n-6) has been shown to enhance mitochondrial fitness, thereby promoting memory differentiation and strengthening anti-tumour immunity^106^. Trans-vaccenic acid — derived primarily from ruminant fats such as dairy products — has also been reported to improve CD8^+^ T cell function by antagonizing GPR43 (G-Protein Receptor 43), a receptor associated with the suppressive effects of short-chain FAs^107^. From an intracellular perspective, TCR activation induces structural remodelling of the acyl chains within phosphoinositides, shifting from polyunsaturated to more saturated forms; maintenance of CD8^+^ T cell anti-tumour activity requires sufficient availability of polyunsaturated phosphoinositides to support this signalling cascade^108^. In addition, ketone bodies such as β-hydroxybutyrate (β-OHB) can undergo intracellular ketolysis to generate acetyl-CoA, which participates in the epigenetic regulation of the IFNγ locus and consequently enhances CD8^+^ T cell effector function^109^.

In contrast, chronic lipid accumulation within the TME often drives pathological metabolic stress that undermines CD8^+^ T cell anti-tumour activity. Competitive uptake of FAs by tumour cells limits nutrient availability and metabolic insufficiency^19,104^, whereas excessive CD36-mediated uptake of oxidized lipids promotes lipid peroxidation, mitochondrial dysfunction and ferroptotic cell death. Other studies demonstrate that PD-1 inhibitory signalling further enforces a metabolic switch from glycolysis towards FAO, resulting in diminished activity of intratumoural CD8^+^ T cells^110,111^. Accumulated cholesterol has also been shown to induce ER stress and promote exhaustion of CD8⁺ T cells^98^. Notably, IL-9-producing cytotoxic T cells (Tc9) display enhanced anti-tumour activity compared with type 1 cytotoxic T cells (Tc1), at least in part by upregulating intracellular cholesterol efflux, thereby alleviating cholesterol-induced stress^112^. In addition to cholesterol itself, its derivatives — including conjugated and secondary bile acids — have been reported to exacerbate oxidative and ER stress in CD8^+^ T cells in HCC, thereby diminishing their anti-tumour function^113^. However, not all cholesterol species exert suppressive effects; membrane-inserted cholesterol, rather than stored cholesteryl esters, has been shown to facilitate TCR clustering and immune synapse formation, ultimately enhancing CD8^+^ T cell activation^114^.

In PDAC, neutral lipids and glycerophospholipids progressively accumulate from early to late disease stages. This environmental shift leads to the dysfunction of intrapancreatic CD8^+^ T cells by attenuating the expression of very long-chain acyl-CoA dehydrogenase (VLCAD), resulting in intracellular VLCFA accumulation and subsequent mitochondrial impairment^18^. Accumulated oxidized lipids and PUFAs within the TME can also be taken up by CD8^+^ T cells through the scavenger receptor CD36, promoting lipid peroxidation that diminishes effector function or triggers ferroptotic cell death^115,116^. PGE_2_ further suppresses CD8^+^ T cell immunity by engaging the EP2/EP4 signalling axis, thereby impairing IL-2-mediated expansion and inducing oxidative-stress-driven death of tumour-infiltrating lymphocytes^117^. Lipid accumulation in the TME is exacerbated under conditions of obesity, which has been shown to reduce CD8^+^ T cell infiltration, trafficking and effector capacity while promoting exhaustion^118^. Dietary stearoyl carnitine (CAR18:0) has been identified as a contributor to these obesity-associated immunosuppressive effects^21^.

Taken together, lipid metabolism does not exert uniform effects on CD8⁺ T cells. Although controlled FAO and selective lipid utilization, including cholesteryl esters, linoleic acid and trans-vaccenic acid, support memory formation and anti-tumour immunity, excessive lipid uptake and peroxidation within the TME drive metabolic stress, exhaustion and loss of effector function.

### Natural killer cells

Natural killer (NK) cells serve as potent innate anti-tumour effectors capable of direct cytotoxicity through antibody-dependent cell-mediated cytotoxicity (ADCC) or cytolytic granule release. In parallel with CD8^+^ T cells, NK cells rely heavily on glycolysis to support their effector functions, yet they are also profoundly influenced by lipids in the tumour TME^119^. NK cells readily take up lipids abundant in the TME or in tumour-derived ascites and store them as intracellular LDs, placing lipid metabolism at a critical regulatory node for NK-cell function.

In most tumour contexts, excessive lipid accumulation drives pathological metabolic reprogramming, which suppresses NK-cell cytotoxicity. Lipid uptake and LD accumulation activate PPARγ signalling, which suppresses glycolytic metabolism and impairs NK-cell effector function^120,121^ (Fig. 3). However, recent studies have proposed that intracellular LDs may also exert a beneficial effect on NK cell anti-tumour cytotoxicity^119,122^, indicating that lipid storage may play a more complex role in NK-cell biology than previously appreciated. In ovarian cancer, NK cells are enriched in malignant ascites compared with blood, primary tumour tissue and the omentum, yet they display markedly reduced cytotoxic activity. This impairment has been attributed to the high abundance of polar lipids — such as phospholipids and ceramides — within the ascitic environment, among which phosphatidylcholine (36:1) is taken up extensively by NK cells. Excessive accumulation of this lipid induces lipid overload and disrupts plasma-membrane order, thereby compromising NK-cell effector function^119^.

Nevertheless, emerging evidence indicates that lipid metabolism in NK cells is not uniformly suppressive but instead depends on tightly regulated, lipid species-specific programmes. Similarly, NK cells derived from patients with B cell lymphoma exhibit diminished activity in the lipid-rich blood-cancer environment, in which excessive lipid accumulation suppresses NK-cell cytotoxicity^123^. In this context, lipid metabolic regulators such as PPARγ have been shown to help to sustain NK-cell function. More recently, studies in both human and mouse NK cells have identified Mef2C as a key transcriptional regulator of NK lipid metabolism. Mef2C controls the SREBP pathway, which is essential for generating the lipids required for NK-cell activation. Notably, supplementation of cholesterol or palmitate failed to rescue the anti-tumour defects of Mef2C-deficient NK cells, whereas oleate supplementation successfully restored their anti-tumour cytotoxicity, underscoring the selective requirement for specific lipid species in NK-cell function^122^.

Collectively, these studies suggest that lipid metabolism in NK cells is largely immunosuppressive in lipid-rich TMEs, but selective control of lipid synthesis and composition — not lipid abundance per se — can preserve NK-cell cytotoxic fitness.

### Regulatory T cells

Tregs play a central role in maintaining immune tolerance by restraining excessive effector-cell activity in autoimmune settings. However, within the TME, Tregs suppress cytotoxic T cell function and thereby reinforce immunosuppression, a process that is further amplified by the accumulation of lipids in the TME. Compared with other immune cells, Tregs undergo metabolic adaptations through multiple mechanisms that protect them from lipid-induced dysfunction, peroxidation and cell death in toxic lipid-rich environments.

Treg cells upregulate CD36 expression, enabling rapid uptake and utilization of lipids. Downstream signalling through CD36 activates PPARβ, which promotes mitochondrial metabolic adaptation and supports both the immunosuppressive function and survival of Treg cells within the TME^124^. Tregs preferentially engage FAO over other metabolic pathways in the lipid-rich TME, which enhances their suppressive capacity^103^. Hence, Treg cells prevent lipid peroxidation, ferroptosis and mitochondrial ROS formation through the activity of glutathione peroxidase 4 (GPX4)^125^ (Fig. 3).

Intratumoural Treg cells undergo metabolic reprogramming, not only to sustain their survival and resilience but also to strengthen their suppressive capacity. In highly glycolytic tumours, lactic acid is taken up by Treg cells through monocarboxylate transporter 1 (MCT1), which has been reported to drive the induction of PD-1 expression^126^. Lim et al. further demonstrated that tumours enhance the suppressive functional fitness of Treg cells by upregulating SREBP, a master regulator of intracellular FA and cholesterol synthesis, and that mevalonate metabolism and protein geranylgeranylation critically contribute to the induction of PD-1 expression^127^. Additional studies have revealed that PD-1 signalling within tumour-infiltrating Treg cells directly regulates their lipid metabolism, thereby enhancing their proliferative capacity and suppressive activity^128^. These findings indicate a tight mechanistic link between PD-1 immune checkpoint signalling and lipid metabolic programming in Treg cells. This concept parallels observations in CD8⁺ T cells, in which PD-1 signalling drives a metabolic shift toward FAO, resulting in diminished anti-tumour activity.

Multiple studies have shown that short-chain FAs, such as butyrate and propionate, enhance the suppressive function of Treg cells in intestinal immune homeostasis and autoimmune diseases including multiple sclerosis. In contrast, the impact of LCFAs on Treg biology remains poorly defined, especially within the TME, where the effects of specific FAs or lipid species on Treg function are still largely unexplored. Further research is therefore needed to clarify how distinct lipid metabolites shape Treg activity in tumours and contribute to immunosuppression^129–131^.

### Tumour-associated macrophages

Macrophages are highly plastic innate immune cells that adapt to diverse microenvironmental cues, contributing not only to antimicrobial and autoimmune responses but also to tumour progression. They constitute one of the most abundant immune-cell subsets within the TME. Although historically categorized into classically activated M1 macrophages (driven by LPS/IFNγ) and alternatively activated M2 macrophages (driven by IL-4/IL-13), TAMs in tumours rarely fall into strictly dichotomous M1/M2 states. Instead, they adopt context-dependent activation states shaped by tumour-derived signals, stromal interactions, and metabolic constraints. From a metabolic perspective, macrophage polarization is tightly linked to substrate utilization. M1 macrophages rely on glycolysis and interrupted TCA-cycle activity to support pro-inflammatory and tumoricidal functions. In contrast, IL-4-driven M2 macrophages preferentially engage FA uptake and β-oxidation, which promotes oxidative metabolism and supports immunoregulatory programmes^132,133^. Within the TME, metabolic rewiring reinforces macrophage skewing towards the M2-like, pro-tumoural phenotype.

Given the central role of macrophage metabolism in regulating their phenotype, targeting metabolic pathways in TAMs has emerged as a potential therapeutic avenue. In ovarian cancer, a metabolically restrictive and cholesterol-rich environment, pharmacological inhibition of cholesterol synthesis with simvastatin has shown promise in overcoming drug resistance^134^. In squamous cell carcinoma and head and neck cancer, macrophages within tumour lesions exhibit prominent M2 polarization, suggesting a therapeutic window for targeting pro-tumoural macrophage programmes^135^. In prostate-cancer models, supplementation with omega-3 FAs suppresses tumour invasion by inhibiting angiogenesis and reversing M2 macrophage-mediated T cell suppression. Mechanistically, these effects are mediated through GPR120-dependent signalling, highlighting the potential of lipid-sensing GPCRs as targets for TAM reprogramming^136^. TAMs exhibit elevated intracellular lipid content compared with bone-marrow-derived macrophages, largely because of enhanced CD36-mediated lipid uptake. These lipids serve as substrates for augmented FAO, which drives STAT6-dependent transcriptional programmes and promotes M2-like, tumour-supportive differentiation^137^. In murine breast-cancer models, M2-like MHCII^–^Ly6C^–^ TAMs express high levels of adipocyte FA–binding protein (A-FABP), which amplifies IL-6/STAT3 signalling and facilitates tumour growth^138,139^. Single-cell transcriptomic profiling of TAMs from patients with prostate cancer reveals widespread dysregulation of lipid metabolic pathways^140^. Notably, TAMs from HFD-fed mice closely recapitulate this transcriptional signature, establishing a direct mechanistic link between dietary lipid intake and TAM functional states. Inhibition of FAO with etomoxir or FAO-targeting siRNAs reduces IL-1β and IL-6 production in M2 macrophages and limits the migratory capacity of hepatocellular carcinoma cells^141^. Other molecular regulators also modulate TAM lipid metabolism. Loss of RIPK3 enhances PPAR pathway activity, driving FAO-dependent M2 polarization in HCC models^142^. In colorectal cancer, single-cell RNA sequencing identified DPP7 as a marker of a TAM subset that maintains FAO through de-ubiquitination of CPT1A, ultimately promoting CD8^+^ T cell exhaustion^143^. The immunomodulatory effects of dietary FAs on macrophages are multifaceted. Oleic acid, for example, enhances iNOS expression yet also promotes M2-like polarization and immunosuppressive activity. Arginase-1 (ARG1), a hallmark of M2 macrophages, further contributes to T cell suppression within the TME^144,145^. PPARγ, a lipid-sensing nuclear receptor, is a key transcriptional regulator of M2 macrophage differentiation and improves systemic metabolic homeostasis by enhancing lipid utilization and insulin sensitivity^146^. High concentrations of linoleic acid and arachidonic acid — both PPAR agonists — drive macrophage polarization towards an M2-like phenotype in tumours, reinforcing immunosuppression and supporting tumour progression^147,148^ (Fig. 3).

## Dietary lipids and tumour progression

### High-fat diets accelerate tumour growth: fatty acid specificity matters

Dietary lipids play a critical role in modulating tumour progression, as demonstrated through extensive preclinical investigations. Early studies established that HFDs promote tumour growth in murine models, with subsequent research revealing that this effect is highly dependent on the specific composition of dietary FAs^149^. Multiple investigations have documented that HFDs induce profound immunological alterations within the TME, resulting in accelerated tumour growth. These changes are particularly evident in CD8^+^ T cells, myeloid-derived suppressor cells, CD4^+^ T cells and Tregs^19,118,150–152^.

Beyond correlative associations between HFDs and cancer risk, a growing body of mechanistic studies have demonstrated that lipid-driven metabolic reprogramming in immune cells can alter anti-tumour immunity and therapeutic responsiveness^19^. For example, lipid-regulated pathways such as SREBP-dependent cholesterol biosynthesis have been shown to reinforce the suppressive fitness of intratumoural regulatory T cells^127^, whereas obesity-associated metabolic cues induce immune checkpoint expression and functional exhaustion in myeloid populations^153^.

Building on these insights, recent advances in the field have delineated the differential effects of specific dietary FAs on tumour biology. Notably, the Lynch group demonstrated that HFDs derived from lard, beef tallow or butter — all rich in saturated FAs — accelerate tumour growth, whereas HFDs based on coconut oil, palm oil or olive oil do not produce equivalent tumour-promoting effects, despite comparable total fat content^21^. These findings underscore that FA composition, rather than total fat intake alone, determines tumour outcomes. This section reviews current understanding of how distinct classes of dietary FAs influence tumour progression.

#### Tumour-promoting lipids

##### Saturated fatty acids promote tumour growth

SFAs are LCFAs characterized by the absence of double bonds in their carbon structure. Palm oil, animal fats (including butter and lard) and similar sources contain high proportions of SFAs, with palmitic acid (C16:0) being predominant. SFAs have been implicated in promoting pro-inflammatory responses within the host^154,155^. In experimental models, SFA-enriched diets have consistently demonstrated tumour-promoting effects. In a diethylnitrosamine-mediated HCC model, palmitic acid enhanced tumour development^156^. Similarly, in prostate-cancer models, SFA-rich HFDs augmented transcription of *MYC*, resulting in increased tumour-cell proliferation^157^. Palmitate not only promotes tumour-cell growth but also impairs anti-tumour T cell immunity. In tumour-bearing mice, palmitate administration induces CD8^+^ T cell exhaustion through disruption of mitochondrial activity, resulting in exacerbation of tumour progression^158^.

##### Omega-6 fatty acids enhance tumour growth

Omega-6 FAs — particularly linoleic acid derived from safflower oil — have been associated with tumour-promoting effects. Supplementation with linoleic acid enhances tumour growth in mammary-tumour models, demonstrating opposing biological activity to its omega-3 counterparts^149^. Linoleic-acid-enriched diets increase PGE_2_ levels and activate the Ras–Raf–ERK signalling cascade in lung carcinogenesis models^159^. At the cellular level, CD8^+^ T cells take up linoleic acid through epidermal FA-binding protein (E-FABP), and subsequent linoleic-acid accumulation induces apoptosis of CD8^+^ T cells in a mitochondrial reactive oxygen species-dependent manner^160^. This mechanism compromises anti-tumour immune surveillance, facilitating tumour progression. Beyond immune-cell dysfunction, in vitro treatment with linoleic acid enhances migration capacity of 4T1 tumour cells through FFAR1 and FFAR4-dependent signalling, resulting in increased brain and lung metastasis^161^. These findings demonstrate that linoleic acid promotes tumour progression through both immunosuppressive and direct tumour cell-intrinsic mechanisms. The tumour-promoting effects of linoleic acid are supported by large-scale epidemiological data. Recent studies demonstrated that mTORC1 is activated in triple-negative breast cancer through FABP5-mediated linoleic acid uptake^162^. Analysis of prospective cohort studies encompassing 787,490 participants revealed that consumption of more than 1 g of linoleic acid per day increases the relative risk of colorectal, rectal and colon cancers, providing compelling evidence for limiting dietary omega-6 FA intake in cancer-prevention strategies^163^.

##### High cholesterol and Western diet exacerbates tumour progression and metastasis

Beyond individual FAs, dietary patterns characterized by high cholesterol intake combined with high sugar and high fat — typified by the Western diet — also promote tumour progression in murine models^164^. Western diet increases plasma cholesterol levels and promotes LDLR expression in tumour tissue, resulting in enhanced tumour growth in PyMT mammary gland tumour models^165^.

Mechanistically, LDLR upregulates SERPINE2 transcription, which is essential for vascular mimicry and intravasation in breast-cancer cells, thereby supporting metastasis^166^. In the ovarian-cancer model, enhanced membrane cholesterol efflux promotes IL-4-mediated macrophage reprogramming, thereby reinforcing pro-tumorigenic TAM phenotypes^167^. Case–control studies in Canada have described positive associations between cholesterol intake and tumour incidence, and meta-analyses demonstrate that dietary cholesterol augments the risk of gastric cancer^168,169^. These findings indicate that cholesterol, independent of FA composition, contributes to tumour progression.

#### Tumour-suppressive lipids

##### Omega-3 fatty acids exhibit tumour-suppressive properties

Omega-3 FAs represent a diverse class of PUFAs with distinct biological properties depending on their source and structure. They are distinguished into marine-derived eicosapentaenoic acid (EPA) and docosahexaenoic acid (DHA), and plant-derived alpha-linolenic acid (ALA).

*Marine-derived omega-3 fatty acids (EPA/DHA):* Fish oil-derived omega-3 FAs have demonstrated consistent tumour-suppressive properties across multiple cancer models. In breast-cancer studies, fish-oil-fed mice exhibited significantly reduced tumour growth and metastasis compared with corn-oil-fed controls^170,171^. Additional investigations have shown that dietary omega-3 supplementation inhibits mammary-tumour growth in GPR120-expressing models through receptor-mediated signalling mechanisms^172^. Mechanistically, dietary supplementation with omega-3 FAs using menhaden oil or golden algae reduces tumour growth in human adenocarcinoma xenograft models through diminished angiogenesis-related signalling, as demonstrated by decreased expression of angiogenic transcripts such as *Vegf*^173^. The anti-tumour effects of omega-3 FAs extend beyond xenograft models to spontaneous tumour development. In the Apc^Min/+^ colon-cancer model, tumour burden is significantly reduced by dietary supplementation of omega-3, an effect potentially mediated through activation of type-1 cannabinoid signalling pathways^174^. Although excessive intake of omega-3 FAs may induce obesity, omega-3 supplementation protects against tumour progression by inducing apoptosis of pro-tumorigenic TAMs^175^. This selective elimination of immunosuppressive myeloid populations represents a key mechanism by which omega-3 FAs reshape the TME. Furthermore, omega-3 diet synergistically reduces tumour growth and metastasis when combined with 5-fluorouracil chemotherapy, while simultaneously reducing the anti-neoplastic side effects of 5-fluorouracil^176^. This chemoprotective effect suggests that omega-3 supplementation may improve the therapeutic index of cytotoxic chemotherapy. At the cellular level, omega-3 diet enhances the tumour-killing capacity of T cells and impairs the TAM polarization into M2 phenotypes^177^. This dual effect — augmenting anti-tumour immunity while reducing immunosuppressive myeloid cells — provides a mechanistic insight into the robust anti-tumour effects observed across multiple preclinical models. This enhanced immune surveillance represents a critical mechanism underlying the tumour-suppressive effects of marine omega-3 FAs and suggests potential clinical applications in combination with cancer immunotherapy.

*Plant-derived alpha-linolenic acid:* ALA, abundant in plant-derived oils such as canola oil, has demonstrated tumour-suppressive effects comparable with gamma irradiation therapy, notably without the harmful side effects associated with radiation treatment^178^. Dietary intake of ALA increases serum levels of ALA, EPA and DHA while simultaneously reducing prostate-cancer growth^179^. In the experimental HCC model, although dietary supplementation of ALA exerts limited effects on tumour growth, it produces synergistic anti-tumour effects when combined with immune checkpoint blockade against HCC by increasing CD8^+^ T cell infiltration into tumour tissue^180^. Several prospective cohort studies provide translational evidence supporting these preclinical findings. Blood levels of ALA correlate inversely with colorectal cancer risk, suggesting that consumption of ALA may represent a potential therapeutic strategy for cancer prevention^181^.

#### Context-dependent lipids

##### Omega-9 fatty acids: the paradox of oleic acid

Oleic acid, the predominant MUFA in olive oil, presents a complex and context-dependent relationship with tumour progression. Classically, oleic acid has been considered relatively tumour suppressive compared with linoleic acid^149,159,182^. However, emerging evidence reveals unexpected pro-tumorigenic properties of oleic acid under specific conditions. Treatment with oleic acid upregulates matrix metalloproteinase-9 (MMP-9) expression in tumour cells, increasing invasiveness through EGFR-Src signalling pathway activation^183–185^. Transcriptomic analysis suggests that dysregulation of AP-1 and MMP-related signalling may contribute to the aggravated tumour burden observed in olive-oil-diet-fed mice^185^. Clinical observations support these experimental findings. Oleic acid is enriched in tumour tissue of patients with obesity and colorectal cancer, and oleic acid-rich diets promote tumour growth comparable with HFDs, with decreased frequency of inflammatory TAMs in an experimental mouse model^186^. These findings challenge the traditional view of olive oil as being uniformly protective against cancer.

##### Clinical evidence: dietary lipid interventions in cancer patients

The effects of dietary lipid modifications on tumour progression have been investigated in human populations for decades, with particular focus on patients with breast cancer. In 1997, Norman and colleagues reported that 2 years of low-fat-diet intervention reduced the area of radiographic features associated with increased breast-cancer risk^187^. Subsequently, large-scale randomized clinical trials have provided more definitive evidence regarding the relationship between dietary-fat modification and cancer outcomes. In randomized clinical trials, reduction of dietary-fat intake led to modest benefits for breast-cancer outcomes. Cancer incidence following death decreased by 35% during the intervention period and by 15% over cumulative follow-up^188–191^. However, when compared with low-fat diets, Mediterranean-diet interventions composed of extra-virgin olive oil resulted in more substantial beneficial effects for the prevention of primary breast cancer^182^. Omega-6 supplementation significantly reduced cancer-related fatigue among breast-cancer survivors, demonstrating benefits beyond tumour control^192^. However, the effects of dietary omega-6 on tumour growth vary considerably among cancer types^163,193^. High ratios of blood circulating omega-6/omega-3 PUFAs are associated with increased risk factors for cancer development^194^. The primary benefit of omega-3 PUFA supplementation appears to be improvement in cancer-associated symptoms, including cachexia, inflammation, neuropathy, post-operative complications and overall quality of life^195^. These palliative benefits represent an important consideration for cancer patient care, even when direct anti-tumour effects may be modest or context dependent (Table 1).

**Table 1:** Summary of dietary fatty acids and their effects on tumour progression.

Fatty acid class	Primary sources	Key examples	Effect on tumour growth	Tumour-intrinsic mechanism	Immune cell-related mechanism	Clinical evidence	Refs.
**SFAs**	Lard, beef tallow, butter, palm oil	Palmitic acid (C16:0)	Pro-tumorigenic	↑ tumour-cell proliferation (*MYC*)	↑ CD8^+^ T exhaustion	–	^156^ ^157^
**Omega-6 PUFA**	Safflower oil, corn oil, soybean oil	Linoleic acid (C18:2n-6)	Pro-tumorigenic	↑ PGE_2_ → Ras-Raf-ERK activation ↑ tumour cell migration and metastasis ↑ tumour proliferation (↑mTORC1)	CD8^+^ T cell apoptosis (↑mito-ROS)	>1 g/day increases colorectal cancer risk (*n* = 787,490)	^159^ ^161^ ^160^ ^162^
**Cholesterol/Western diet**	High-fat, animal products	Cholesterol	Pro-tumorigenic	↑ tumour metastasis (LDLR → ↑ SERPINE2)	Cholesterol efflux — ↑ pro-tumorigenic TAMs	Positive association with gastric cancer Increased tumour incidence (Canadian case–control)	^165^ ^166^ ^169^ ^168^
**Omega-3 PUFA (marine)**	Fish oil, menhaden oil, golden algae	EPA (C20:5), DHA (C22:6)	Anti-tumorigenic	GPR120-mediated signalling → tumour suppression; ↓ angiogenesis	↑ CD8^+^ T, ↓ pro-tumorigenic-TAM (apoptosis) in TME	Improves cachexia and quality of life	^172^ ^173^ ^175^ ^177^ ^195^
**Omega-3 PUFA (plant)**	Canola oil, flaxseed	ALA (C18:3n-3)	Anti-tumorigenic	↓ prostate cancer growth	Synergy with checkpoint blockade; ↑ CD8^+^ T in TME	Inverse correlation with colorectal cancer risk	^179^ ^180^ ^181^
**Omega-9 MUFA**	Olive oil, avocado	Oleic acid (C18:1n-9)	Context-dependent (paradoxical)	Anti-tumour: ↓ tumour growth (↓ERK) Pro-tumour: ↑ tumour migration (MMP9-EGFR-Src)	Context-dependent effects on macrophage polarization	Mediterranean diet superior to low-fat for breast-cancer prevention Enriched in obese CRC tumours	^159^ ^182^ ^186^

ALA, alpha-linolenic acid; CRC, colorectal cancer; DHA, docosahexaenoic acid; EGFR, epidermal growth factor receptor; EPA, eicosapentaenoic acid; LDLR, low-density lipoprotein receptor; mito-ROS, mitochondrial reactive oxygen species; MMP-9, matrix metalloproteinase-9; MUFA, monounsaturated fatty acid; PGE_2_, prostaglandin E_2_; PUFA, polyunsaturated fatty acid; SFA, saturated fatty acid; TAM, tumour-associated macrophage; TME, tumour microenvironment.

## Concluding remarks

Cancer cells reprogram their lipid metabolic pathways to sustain survival and rapid proliferation within the harsh TME, driven by oncogenic activation, loss of tumour suppressor genes, and dynamic interactions with neighbouring cellular components. Tumour-infiltrating immune cells, stromal cells and adipocytes also undergo lipid metabolic alterations, a process further amplified by cancer-cell-derived cytokines and lipid mediators, ultimately reshaping the cellular composition and functional phenotypes within the TME. This interconnected lipid metabolic network contributes to the establishment of an immunosuppressive TME and plays a critical role in tumour progression and therapeutic responses. Therefore, a detailed mechanistic understanding of lipid metabolic reprogramming in cancer cells and their metabolic crosstalk with neighbouring cells is essential for the development of effective cancer treatments.

Membrane lipid composition that varies in cancer cells has been shown to determine selective dependencies on SCD or GPX4 (ref. ^5^). Cancer cells enriched in saturated FAs or MUFAs exhibit heightened susceptibility to ER-stress-induced cell death and therefore display a strong dependence on SCD activity. In contrast, cancer cells with membrane lipids enriched in PUFAs require indispensable GPX4 expression to prevent ferroptosis, thereby exposing distinct therapeutic vulnerabilities. Together, these findings highlight that membrane lipid composition is not merely a structural feature but a critical determinant of metabolic dependency in cancer cells, providing a basis for therapeutic strategies to selectively target cancer subtypes with distinct lipid-dependent states.

## Outlook

A key challenge in cancer lipid metabolism is defining context-specific dependencies that shape tumour progression and anti-tumour immunity. Unresolved questions include how lipid utilization programmes vary across tumour types and disease stages, and how tumour lipid metabolism can be selectively targeted without impairing immune-cell function. This complexity is driven by tumour metabolic plasticity, as well as intratumoural heterogeneity and spatial variation in lipid availability within the TME, which collectively contributes to adaptive resistance to single-agent metabolic therapies.

Recent advances in high-resolution spatial lipidomics, together with single-cell and integrative multi-omics approaches, and in vivo functional perturbation strategies, now enable comprehensive profiling of FA composition within tumours, uncover context-dependent metabolic vulnerabilities and clarify tumour–immune metabolic crosstalk. Ultimately, these insights will inform rational combinatorial strategies that effectively disrupt tumour lipid metabolism while enhancing immune-cell function, thereby improving the efficacy of current cancer therapies.
